# Suppurative Thyroiditis: Coinfection by Nocardia spp. and Mycobacterium tuberculosis in an Immunocompromised Patient

**DOI:** 10.7759/cureus.65744

**Published:** 2024-07-30

**Authors:** Jhon Edwar Garcia Rueda, Andrés David Monsalve Naranjo, Carolina Giraldo Benítez, Juan David Ramírez Quintero

**Affiliations:** 1 Internal Medicine, Pontifical Bolivarian University, Medellin, COL; 2 Internal Medicine, Pablo Tobon Uribe Hospital, Medellin, COL

**Keywords:** mycobacterium tuberculosis, thyroiditis, tuberculosis, thyroid, nocardiosis

## Abstract

Suppurative thyroiditis is a rare entity with a low incidence in thyroid diseases, manifesting with pain, fever, dysphagia, and dysphonia. Its infrequency is explained by the thyroid gland's resistance to infections due to its encapsulated position, high blood flow, bactericidal action of iodine, and extensive lymphatic network.

We present the first report in the literature of a 72-year-old woman with a history of inflammatory myopathy and immunosuppression diagnosed with suppurative thyroiditis co-infected with Nocardia spp. and Mycobacterium tuberculosis. This entity requires a high clinical suspicion, and fine-needle aspiration biopsy (FNAB) is preferred as the diagnostic method for microbiological sampling. Although rare, it carries high morbidity and mortality if not suspected in time.

## Introduction

Nocardiosis is an uncommon bacterial infection. It was described by veterinarian Edmon Nocard in 1888 when he identified an acid-fast Gram-positive microorganism causing disease in cattle. A year later, Vittore Trevisan named this genus Nocardia after Nocard, which makes it the first human pathogenic actinobacterium described in the literature [1].

It is acquired from the environment through ingestion, inoculation, and inhalation, the latter being the most common form. Its main characteristic is the formation of abscesses and chronic progression with relapses despite adequate treatment. Its diagnosis requires a high index of suspicion and its identification in cultures is necessary to confirm it. It can spread hematogenously to any organ with special tropism to the central nervous system and infrequently to the thyroid [2].

Thyroid infections are rare since it is a resistant organ due to its encapsulated position, high blood flow, and bactericidal action of iodine and hydrogen peroxide. They occur mainly in immunocompromised patients, with Nocardia mainly affecting those with cellular immunity deficits [2,3]. Mycobacterium tuberculosis is a recognized pathogen in immunocompromised patients; however, infections in the thyroid gland are infrequent, even in areas where it is endemic [4]. We present the first report in the literature of an immunocompromised patient with suppurative thyroiditis due to Nocardia spp. and Mycobacterium tuberculosis.

This article was previously posted to the Research Square preprint server on August 28, 2023.

## Case presentation

A 72-year-old woman, homemaker, and ex-smoker, with a medical history of arterial hypertension and osteoarthritis, consulted a rheumatology clinic for a seven-month history of pelvic girdle weakness and dysphagia. Rheumatology requested studies and confirmed inflammatory myopathy with a risk of bronchial aspiration, so she received three pulses of methylprednisolone at a dose of 500 mg every 24 hours, with subsequent clinical improvement. She was discharged with prednisolone 50 mg per day, methotrexate 12.5 mg every week, and hydroxychloroquine 200 mg per day.

Five months later, she visited the hospital for the appearance of a painful right thyroid nodule, and 15 days later, the appearance of 3 erythematous nodular lesions: on the right twelfth costal arch of 23*12 mm, left thigh of 13*5 mm, and on the right buttock of 10 mm measured by ultrasound. She received 7 days of amoxicillin/clavulanate 875/125 mg every 8 hours with partial improvement of the cutaneous lesions.

**Figure 1:**
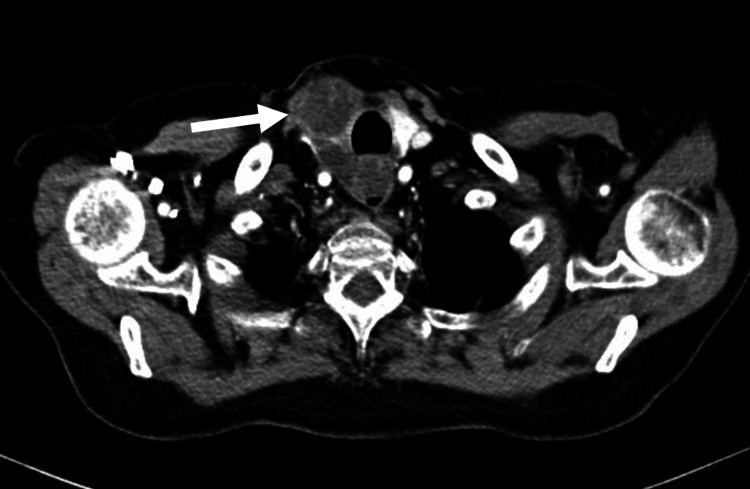
Contrast tomography of the neck A lesion in the right thyroid lobe with a hypodense center extending to the retrotracheal mediastinum

In addition, she started complaining of neuropsychiatric alterations: disorientation, bradypsychia, and right hemiparesis associated with a cough with greenish expectoration and unintentional weight loss of 10 kg, for which she was hospitalized. On admission, thyroid ultrasound documented the right thyroid lobe completely occupied by an irregular nodule; a neck CT scan showed a lesion dependent on the right thyroid lobe extending to the retrotracheal mediastinum up to 3 cm cephalic to the carina (Figure 1); thyroid scintigraphy reported a right cold nodule and the thyroid profile was thyroid-stimulating hormone (TSH): 0.34 µUI/mL, free T4: 1.03 ng/dl, and T3: 51.26 pg/ml.

**Figure 2:**
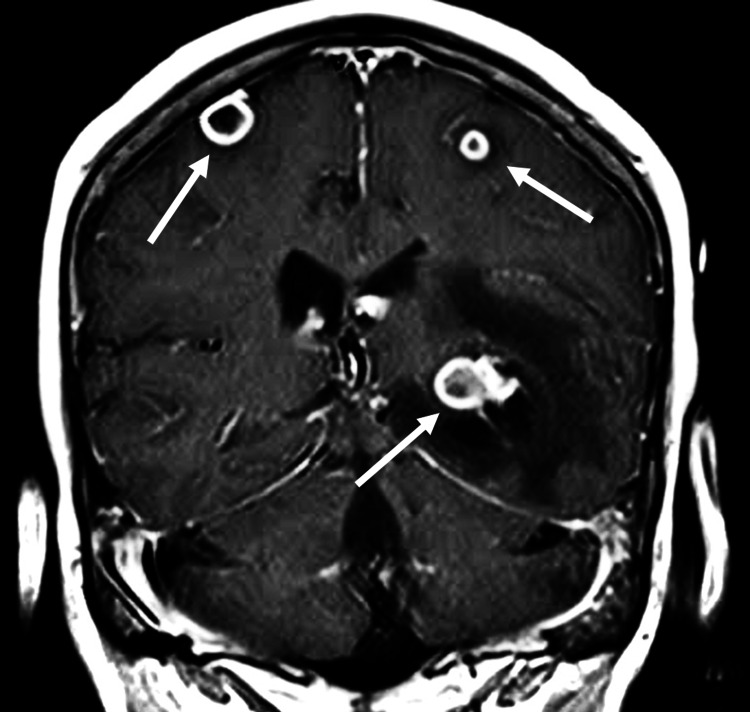
Cranial MRI coronal section Focal hypointense lesions in T1 with annular enhancement after contrast administration, involving both frontal and left temporal lobes

During hospitalization, she developed seizures. A cranial tomography showed multiple intraparenchymal abscesses in the frontal and left temporal lobes (Figure 2). She was transferred to the special care unit.

**Figure 3:**
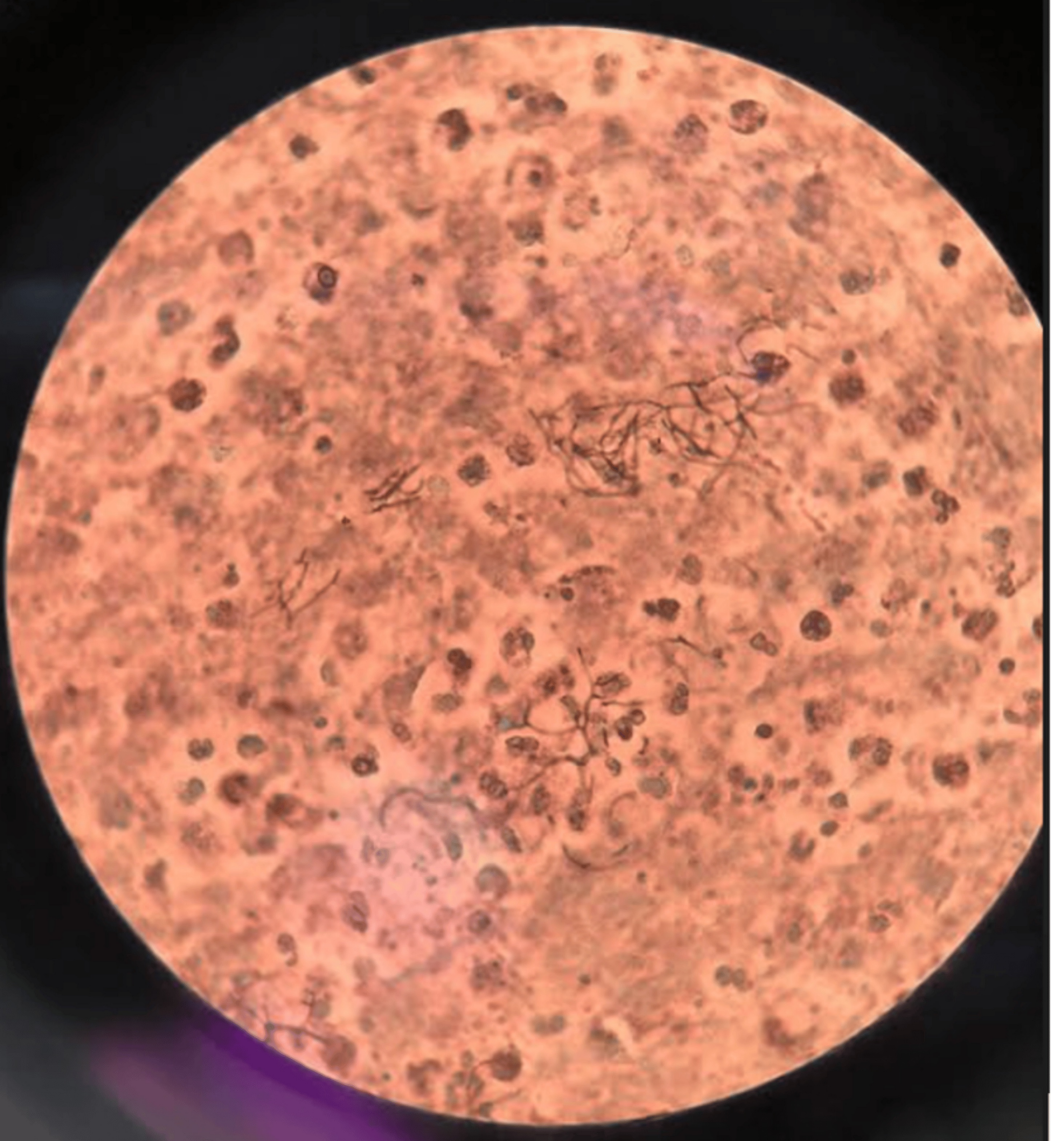
Thyroid biopsy, methenamine silver staining Bifurcated filamentous structures compatible with nocardia spp.

Ultrasound-guided fine-needle biopsy of the thyroid was performed, and pus was obtained and sent for studies. The acid-fast stain was negative, methenamine silver stain performed on the thyroid biopsy showed bifurcated filamentous structures compatible with Nocardia spp. (Figure 3), for which intravenous antibiotic treatment for disseminated nocardiosis was started for 8 weeks with trimethoprim/sulfamethoxazole 320/1600 mg every 8 hours, Amikacin 350 mg every 12 hours, and Imipenem/Cilastatin 500 mg every 6 hours. Subsequently, the growth of acid-fast bacilli compatible with Mycobacterium tuberculosis was reported after 31 days of incubation of the pus sample obtained by fine-needle aspiration (FNA) and antituberculosis treatment was started with a four-drug “HREZ” fixed-dose combination with rifampicin 150 mg, isoniazid 75 mg, pyrazinamide 400 mg, and ethambutol 275 mg; 3 tablets of the HREZ daily.

The patient was also found to have pulmonary nodules of random distribution by simple chest tomography. She underwent bronchoscopy with bronchoalveolar lavage, and the skin biopsy showed large areas of abscessation and multinucleated foreign body giant cells. The acid-fast stain was negative, the aerobic culture and drainage of CNS abscess were negative, and in no other sample was the isolation of Nocardia spp. or M. tuberculosis achieved.

Once the treatment was finished for eight weeks, she was discharged and continued the antituberculosis treatment with doxycycline 100 mg every 12 hours and trimethoprim/sulfamethoxazole 320/1600 mg every 8 hours for 12 months. Four months later, she was readmitted due to clinical deterioration with neurological worsening. She presented a massive episode of bronchial aspiration with secondary aspiration pneumonia, refractory septic shock, multisystem failure and, finally, she died.

## Discussion

The thyroid is an organ resistant to infections due to its encapsulated position that protects it from the outside, high vascularization, lymphatic drainage, high concentration of tissue iodine, and production of hydrogen peroxide for the synthesis of thyroid hormone [3]. The above explains why infectious thyroiditis is infrequent. The exact incidence is unknown, but it is estimated to correspond to 0.1% to 0.7% of thyroid diseases [5].

Suppurative thyroiditis (ST) develops in patients with pre-existing risk factors, especially immunosuppression states, and on a thyroid that may be previously healthy, as demonstrated in the series of Lafontaine et al., published in 2021, in which 200 cases of ST were collected between 2000 and 2020 and in which only 20% of the 130 patients who had bacterial ST had pre-existing thyroid disease [3,6].

Clinically, thyroiditis manifests with pain (89-100%), fever (82-92%), dysphagia (46-91%), erythema (38-82%), and dysphonia (15-82%) as main symptoms [6,7]. In the case presented, the patient presented pain as the only symptom associated with thyroiditis. Within the laboratory findings, there are no specific paraclinical findings to guide toward a possible ST, as any infection can present with elevation of acute phase reactants and the thyroid profile can be variable, Yu et al. published a series of 191 cases reported between 1980 and 1997 in which 68% of the patients were euthyroid [8]; however, the most recent meta-analysis by Lafontaine found that 42% of bacterial ST (BST) and 40% of fungal ST (FST) had hyperthyroidism at presentation and that at least 36% of BST cases had free T4 more than twice the upper limit of normal. In the long term, 21% of the BST and 50% of the FST developed hypothyroidism, which makes it difficult, only by thyroid tests, to differentiate between ST and subacute thyroiditis (SAT) [6]. Our patient did not have a TSH measurement during the course of thyroiditis.

In relation to imaging studies, ultrasound and tomography can be very nonspecific in the early stages since abscess formation can be observed in acute inflammation and thyroid scintigraphy is usually abnormal, with evidence of cold nodules [9,10]. This is why FNA is preferred as the method of choice for the diagnosis of ST since it allows not only the collection of microbiological samples but also the performance of therapeutic drainage and differentiation with SAT in cases of thyrotoxic presentation [3,11].

Regarding etiology, in the three large series we have so far, infection by Gram-positive bacteria, mainly Streptococcus spp. (9-16%) and Staphylococcus spp. (9.5-15%), occurred more frequently, while Nocardia spp. was documented in only 6 patients in the 3 series, representing 4% in the largest series. Among the Gram-negative microorganisms, Salmonella was the most frequently isolated microorganism. M. tuberculosis had a prevalence between 9.3-16%; it is noteworthy that 11% of the STs in Lafontaine's series were polymicrobial [6-8].

Regarding infection by Nocardia, it is known that in 60% of cases, there is a pre-existing compromise of the immune system and that having received high doses of prednisone has an odds ratio (OR) of 26 for infection [12,13].

The clinical presentation is variable, there may be involvement limited to the skin, pulmonary involvement occurs in 73% of patients with subacute or chronic symptoms, characterized by cough, dyspnea, hemoptysis, and fever. Abscess formation and cavitary disease may occur, in some cases endobronchial masses and empyema [14,15]. In the case of central nervous system (CNS) involvement, this manifests as granulomas or abscesses, which in 50% of the cases are multiple, and it is known that when there is CNS involvement in 80% of the cases as presented in ours, the patients are immunosuppressed, and this will define prognosis since an immunosuppressed patient with nocardiosis in the CNS has a mortality of 55%. In rarer cases, there may be keratitis, bacteremia, and disseminated presentation characterized by the formation of generalized abscess foci in two or more sites is not uncommon [16-18].

Regarding ST by Nocardia spp., so far we have a series published in 2021 by Esnault et al., of 11 cases documented since 1978, in which all patients were immunosuppressed, 10 of them had disseminated infection, 7 had an infection by N. asteroides, 2 by N. farcinica, 1 by N. brasiliensis, and 1 by N. abscessus, all with variable clinical presentation, from an asymptomatic course, presence of painful cervical nodules, to painful thyromegaly, with dysphagia, dysphonia, and thyrotoxicosis [19].

Diagnosis can be difficult because although it can grow in usual culture media, its best performance is in selective culture media; it requires an incubation time of up to 14 days, and molecular tests, such as PCR, have a specificity of only 74% and do not differentiate infection or colonization [14,20]. In the case presented, isolation of Nocardia was only achieved in the thyroid; it should be noted that the patient had previously taken amoxicillin/clavulanate, which is active against some species of Nocardia, which could have altered the performance of the cultures.

Mycobacterium tuberculosis is a recognized pathogen in patients with immunosuppression; however, infections in the thyroid gland are infrequent, even in areas where it is endemic, it is estimated to occur with a frequency of 0.1-0.4% of all cases of TB. There are few case reports and series of tuberculous thyroiditis, where a predominance in the female sex is reported with a range between 14-83 years and a mean age of 43+17 years for women. Its range of presentation is varied as a solitary nodule or diffuse or multinodular goiter [20].

Regarding the coinfection of Nocardia and tuberculosis, this is the first case reported in the literature, it is an infrequent pathology, due to uncommon germs that coincided in a host with multiple risk factors for infection by both microorganisms, with an unfavorable prognosis.

## Conclusions

Suppurative thyroiditis is a rare entity given the resistance of the thyroid to infections. It develops in patients with risk factors, such as immunosuppression states, especially when there is a deficit of cellular immunity. For this entity, it is required to have high clinical suspicion and as a diagnostic method, FNA is preferred for microbiology sampling. The purpose of this report is to inform the clinician about a rare entity with high morbidity and mortality if it is not suspected on time.
